# Real-world evidence and optimization of vocal dysfunction in end-stage renal disease patients with secondary hyperparathyroidism

**DOI:** 10.1038/s41598-020-79810-0

**Published:** 2021-01-12

**Authors:** Geng-He Chang, Fong-Fu Chou, Ming-Shao Tsai, Yao-Te Tsai, Ming-Yu Yang, Ethan I. Huang, Hui-Chen Su, Cheng-Ming Hsu

**Affiliations:** 1 Department of Otolaryngology – Head and Neck Surgery, Chiayi Chang Gung Memorial Hospital, No 6, Sec. West, Jiapu Rd., Puzi-City, Chiayi County, Taiwan; 2 Health Information and Epidemiology Laboratory, Chang Gung Memorial Hospital, Chiayi, Taiwan; 3 Graduate Institute of Clinical Medical Sciences, College of Medicine, Chang Gung University, Taoyuan, Taiwan; 4 Department of General Surgery, Kaohsiung Chang Gung Memorial Hospital, Kaohsiung, Taiwan; 5 School of Medicine, College of Medicine, Chang Gung University, Taoyuan, Taiwan; 6 Department of Neurology, National Cheng-Kung University Hospital, Tainan, Taiwan

**Keywords:** Health care, Respiratory tract diseases, Chronic kidney disease

## Abstract

Patients with end-stage renal disease (ESRD) may demonstrate secondary hyperparathyroidism (SHPT), characterized by parathyroid hormone oversecretion in response to electrolyte imbalance (e.g., hypocalcemia and hyperphosphatemia). Moreover, this electrolyte imbalance may affect vocal cord muscle contraction and lead to voice change. Here, we explored the effects of SHPT on the voices of patients with ESRD. We used data of 147,026 patients with ESRD from the registry for catastrophic illness patients, a sub-database of Taiwan National Health Insurance Research Database. We divided these patients into 2 groups based on whether they had hyperparathyroidism (HPT) and compared vocal dysfunction (VD) incidence among them. We also prospectively included 60 ESRD patients with SHPT; 45 of them underwent parathyroidectomy. Preoperatively and postoperatively, voice analysis was used to investigate changes in vocal parameters. In the real-world database analysis, the presence of HPT significantly increased VD incidence in patients with ESRD (*p* = 0.003): Cox regression analysis results indicated that patients with ESRD had an approximately 1.6-fold increased VD risk (*p* = 0.003). In the clinical analysis, the “jitter” and “shimmer” factors improved significantly after operation, whereas the aerodynamic factors remained unchanged. In conclusion, SHPT was an independent risk factor for VD in patients with ESRD, mainly affecting their acoustic factors.

## Introduction

Patients with end-stage renal disease (ESRD) may have impaired calcium, phosphorus, and vitamin D metabolism, rendering their kidneys unable to filter out plasma phosphorus produced by the body and to synthesize sufficient vitamin D (particularly calcitriol, the active form of vitamin D)^1–3^. Without treatment, this condition progresses to hypocalcemia, resulting in excessive intact parathyroid hormone (iPTH) secretion by the parathyroid glands and subsequent hypertrophy of these glands; this condition is called secondary hyperparathyroidism (SHPT)^4–6^. Moreover, aforementioned abnormal metabolic complications can induce excessive bone mineral loss and extraskeletal calcification, both of which are associated with increased risks of bone fractures, heart disease, and even death^7–9^.

The clinical symptoms of SHPT include bone pain, malaise, and pruritus^10,11^. SHPT-associated electrolyte imbalance can lead to neuromuscular manifestations, such as skeletal muscular weakness^12,13^; in some cases, weakness of the fine muscles of the vocal cords may occur. Based on our clinical observation, we speculate that voice change is an SHPT-associated symptom.

According to our clinical observations, patients with SHPT often complain of hoarseness and vocal symptoms. This observation, not reported thus far, warrants investigation given its significance for clinicians (particularly otolaryngologists) in the expansion of the disease spectrum and consideration of SHPT in the differential diagnosis of vocal dysfunction (VD). The possible causes of VD in patients with ESRD include dehydration of the vocal fold mucosa or weakness of the vocal fold muscle. In patients with ESRD, dehydration is the predominant cause of VD. In this study, video strobe laryngoscopy (VSL) and voice analysis were performed on days between dialysis to minimize dehydration. Electrolyte imbalance, which was reported in patients with ESRD, may affect voice production^14^.

Therefore, we performed a real-word data analysis using a nationwide database of Taiwan to investigate whether ESRD patients with SHPT have VD risks. Then, we prospectively recruited ESRD patients with SHPT and analyzed their vocal characteristics through VSL and voice analysis before and after parathyroidectomy and investigated vocal motion abnormalities due to SHPT. The aim of this study is to investigate voice changes in patients with SHPT and voice recovery after the surgical procedure. Our findings can help the clinician to evaluate vocal symptoms when diagnosing SHPT.

## Methods

### Part I–real-world data study

We used data from Taiwan’s National Health Insurance (NHI) Research Database (NHIRD). The NHI program, implemented by Taiwan’s government in 1995, had covered almost all Taiwan residents by 2017^15–18^. Subsequently, Taiwan National Health Research Institutes had constructed NHIRD for research use. NHIRD contains NHI beneficiaries’ medical records, including disease diagnosis at outpatient visits and hospitalizations, drug types and dosages, examination items, operation contents, medical expenditure, area of residence, and income level^19^. In the NHIRD, diagnosis coding follows the International Classification of Diseases, Ninth Revised Edition, Clinical Modifications (ICD-9-CM)^20,21^. The NHI system defines chronic kidney failure with long-term renal dialysis as a “catastrophic illness.” The concerned patients are registered in the Registry for Catastrophic Illness Patients (RFCIP)^20^ after audit and are given catastrophic illness–related treatment at subsidized rates^22^. However, the audit process is rigorous, with regular dialysis treatment for ≥ 3 months as an eligibility criterion. Therefore, RFCIP data were used for identifying and recruiting chronic renal failure patients requiring kidney dialysis.

**Figure 1:**
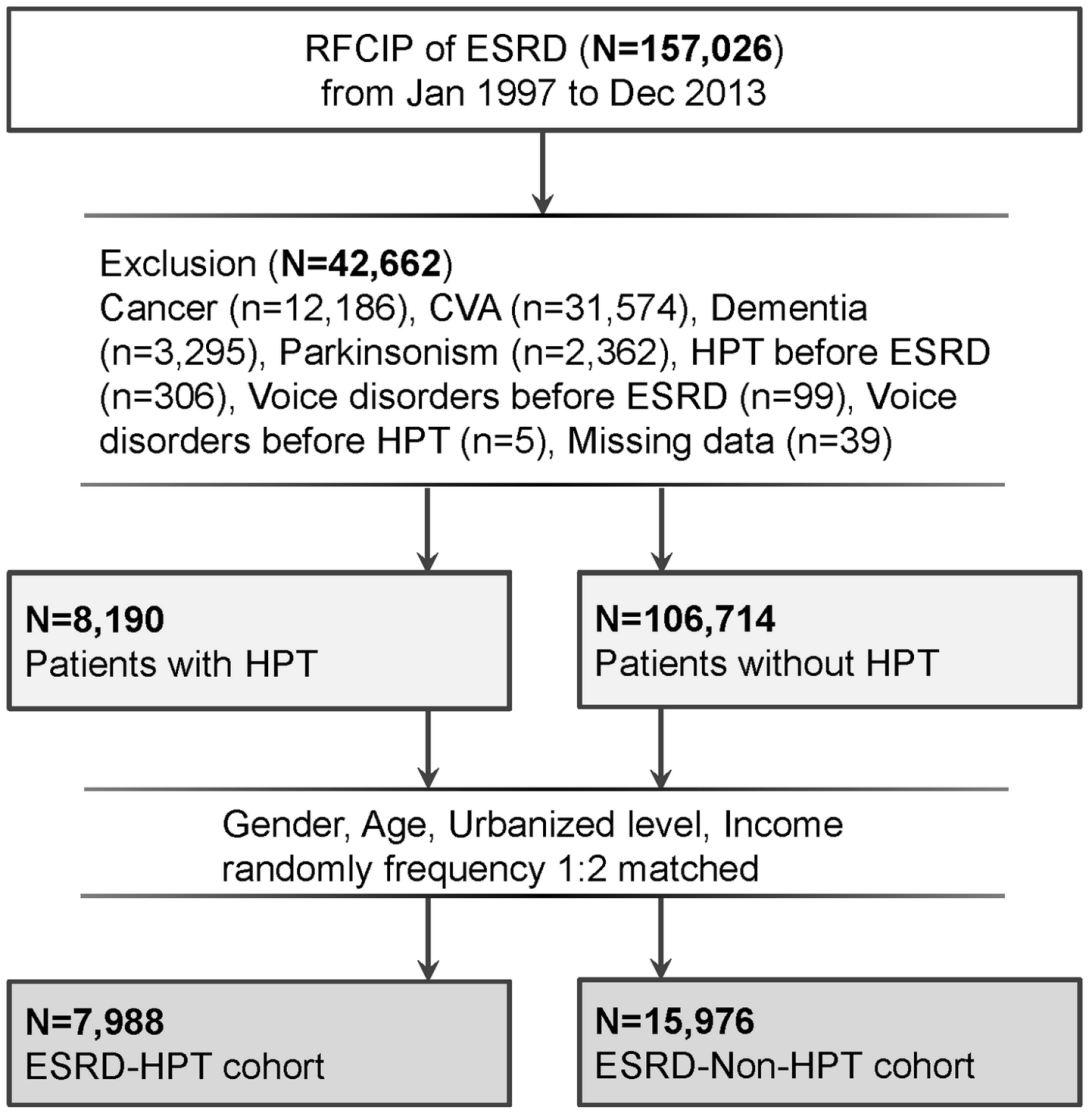
Flow of patient enrolment. RFCIP, Registry for Catastrophic Illness Patients; ESRD, end-stage renal disease; HPT, hyperparathyroidism.

Data of patients with a diagnosis of ESRD between January 1997 and December 2013 were retrieved from the RFCIP by using ESRD-associated ICD-9-CM codes: 585, 586, 403.01, 403.11, 403.91, 404.02, 404.03, 404.12, 404.13, 404.92, and 404.93 (Fig. 1)^20^. Consequently, data of 147 026 patients with ESRD were extracted from RFCIP after the exclusion of patients with comorbid cancer, cerebrovascular accident, dementia, parkinsonism ESRD, and any voice-related diseases before ESRD and HPT. The index date for the study cohort was the HPT diagnosis date.

ICD-9-CM codes of HPT were employed to divide the included patients into the ESRD-HPT (study) and ESRD-non-HPT (comparison) cohorts. Then, all ESRD patients with HPT were matched 1:2 by sex, age, urbanization level, and income level with ESRD patients without HPT, who then formed the comparison cohort. An index date matching that of the study cohort was allocated to the comparison cohort.

The main outcome here was VD occurrence (ICD-9-CM 478.5, 478.7, and 784.5). Both cohorts were followed from the index date to VD diagnosis, death, or December, 2013, whichever occurred first.

Comorbidities were defined using the ICD-9-CM codes noted in the claims data: diabetes mellitus (DM; ICD-9-CM 250), hypertension (HTN; ICD-9-CM 401-405), chronic obstructive pulmonary disease (COPD; ICD-9-CM 491, 492, 494, and 496), liver cirrhosis (LC; ICD-9-CM 571.2, 571.5–571.6), asthma (ICD-9-CM 493), chronic rhinosinusitis (CRS; ICD-9-CM 473) and gastroesophageal reflux disease (GERD; ICD-9-CM 530.11 and 530.81)^23–26^. A comorbidity was included if it appeared in at least 1 inpatient diagnosis or at least 3 outpatient diagnoses.

### Part II–clinical study

For the clinical study, we prospectively recruited patients with SHPT undergoing hemodialysis. The exclusion criteria were past history of vocal mucosal lesion (e.g., nodules or polyps) and vocal fold paralysis as well as current diagnoses of other vocal etiologies (e.g., severe asthma) or other systemic diseases. Of the enrolled hemodialysis patients with SHPT, patients with had refractory symptomatic SHPT received total parathyroidectomy and autotransplantation, which were successful.

The vocal functions within 2 weeks before surgery and 3 months after surgery were documented through VSL on a stroboscopy system (Model 9400) from KayPENTAX (Lincoln Park, NJ, USA). VSL and voice analysis were performed on a day between dialysis sessions. For analysis of the laryngeal image, the following data were considered: absence/presence of vocal fold lesion; wave of vocal fold mucosa; pattern of glottal closure and any involvement of supraglottic structures. The reports from laryngeal examination before and after surgery of each patient were taken from two laryngologists. If there was any difference between two reports from two laryngologists, reevaluation was necessary.

We measured acoustic parameters including jitter, shimmer, mean fundamental frequency (F0), and noise-to-harmonics ratio (NHR) by using a Computerized Speech Laboratory (CSL) system (Model 4500; KayPentax, Lincoln Park, NJ, USA)^27,28^. The participants were asked to phonate the sustained vowel /a/ at their habitual pitch and comfortable loudness, after inhaling deeply. The acoustic parameters of the voice, including fundamental frequency (Hz), jitter (%), shimmer (dB), and noise-harmonic ratio (NHR; dB), were evaluated. The phonation time was 3 s and a segment from the middle of the vowel phonation was analyzed, and bad voice quality data was discarded. Here, F0 is the average of all extracted fundamental frequency values. Shimmer (%) was defined as the average absolute difference between peak amplitudes of consecutive periods divided by the average peak amplitudes during the phonatory segment. Jitter (%) was defined as the average absolute difference between successive periods divided by the average period duration. Moreover, here, the NHR reflected the relative spectral energy contributions of the noise and harmonic components of the acoustic voice signal^29^.

Moreover, aerodynamic parameters including maximum phonation time (MPT) and durations of the sounds “s” and “z” were analyzed by a speech pathologist using a stopwatch^27^. Here, the sound “s” is voiceless (i.e., produced without vibration of the vocal folds), whereas the sound “z” is a voiced counterpart of the sound “s”^30^. The voice of patients who underwent parathyroidectomy was analyzed 1 or 2 weeks before surgery and then 3 months after surgery.

Preoperative and postoperative perceptual evaluation was performed using the GRBAS scoring system (G = grade, R = roughness, B = breathiness, A = asthenia, and S = strain; 0 = normal, 1 = mild, 2 = moderate, and 3 = severe). The GRBAS-sum is the summation of each GRBAS score, and reevaluation was required if a difference of more than 2 points was recorded between the two GRBAS scores. A speech pathologist and an otolaryngologist analyzed the above-mentioned voice parameters in a double-blinded manner. All participants in this study gave written informed consent prior to study begin. The experiment was conducted in line with the relevant guidelines and regulations.

Both parts of this study were approved by the Institutional Review Board of Chang Gung Memorial Hospital (IRB No. 201800401B0).

### Statistical analysis

For Part I of this study, we compared the comorbidities and demographic characteristics of the study and comparison cohorts; here, we used Pearson’s chi-square test and an unpaired Student *t* test to compare categorical and continuous variables, respectively^31^. Next, we included control variables such as sex, age, income level, urbanization level, and comorbidities as covariates in our univariate model to perform a univariate analysis^32^. The variables with *p* < 0.1 in the univariate analysis were included in the multivariate analysis. The cumulative incidence of VD in the 2 cohorts was estimated using a Kaplan–Meier analysis, and then, a two-tailed log-rank test was used determine the relevant differences between the cohorts. We also used multivariable Cox proportional hazard regression models to measure the hazard ratios (HRs) and corresponding 95% CIs for VD. The stability of HRs was also examined using subgroup and sensitivity analyses so as to evaluate whether the comorbidity–SHPT interaction effects on VD were significant.

For Part II of this study, paired *t* and Wilcoxon signed-rank tests were used to analyze the statistical significance of our parametric and nonparametric measurement data, respectively^28^. Pearson correlation coefficient analysis was also applied^28^.

The online version contains supplementary material available at 10.1038/s41598-020-79810-0. All statistical analyses were performed using SAS (version 9.4; SAS Institute, Cary, NC, USA); and significance level was set at *p* < 0.05.

## Results

### Part I–real-world data study

After the 1:2 matching of sex, age, urbanization level, and income level, the study and comparison cohorts contained 7988 patients with HPT (mean ± standard deviation [SD] follow-up period: 4.6 ± 3.3 year) and 15,976 patients without HPT (mean ± SD observation period: 4.5 ± 3.3 year), respectively (Table 1). The study cohort had a significantly higher DM, COPD, CRS, and GERD prevalence than did the comparison cohort. VD incidence rate was also significantly higher in the study cohort (*p* = 0.003; 2.2 vs 1.4 per 1000 person-years). The incidence rate ratio of VD thus was 1.56 (95% CI 1.17–2.09; *p* = 0.003), and the mean duration from HPT diagnosis to VD development was 2.9 ± 2.3 years.

**Table 1:** Demographic Characteristics of the Study and Comparison Cohorts.

Characteristic	ESRD-HPT		ESRD-Non-HPT			*p* value
	N	%	N		%	
Total	7798		15,976			
**Sex**						1.000*
Male	3176	39.8	6352		39.8	
Female	4812	60.2	9624		60.2	
**Age (y)**						1.000*
< 65	6148	77.0	12 296		77.0	
≥ 65	1840	23.0	3680		23.0	
**Urbanized level**						1.000*
1 (City)	2320	29.0	4640		29.0	
2	3837	48.0	7674		48.0	
3	1116	14.0	2232		14.0	
4 (Village)	715	9.0	1430		9.0	
**Income**^**†**^						1.000*
0	1483	18.6	2966		18.6	
1-15 840	1259	15.8	2518		15.8	
15 841-25 000	3769	47.2	7538		47.2	
≥ 25 001	1477	18.5	2954		18.5	
**Comorbidities**						
DM	2729	34.2	8875		55.6	< .001**
HTN	7491	93.8	15 031		94.1	.347**
COPD	1455	18.2	2650		16.6	.002**
Liver cirrhosis	596	7.5	1300		8.1	.068**
Asthma	843	10.6	1628		10.2	.384**
CRS	324	4.1	558		3.5	.029**
GERD	2031	25.4	3331		20.9	< .001**
**Outcome**						
VD	82	2.2	102		1.4	.003**

ESRD, end-stage renal disease; HPT, hyperparathyroidism; DM, diabetes mellitus; HTN, hypertension; COPD, chronic obstructive pulmonary disease; LC, liver cirrhosis; CRS, chronic rhinosinusitis; GERD, gastroesophageal reflux disease; VD, vocal dysfunction.

*Pearson chi-squared test; **Student *t* test; ^**†**^New Taiwan Dollar, per month.

**Figure 2:**
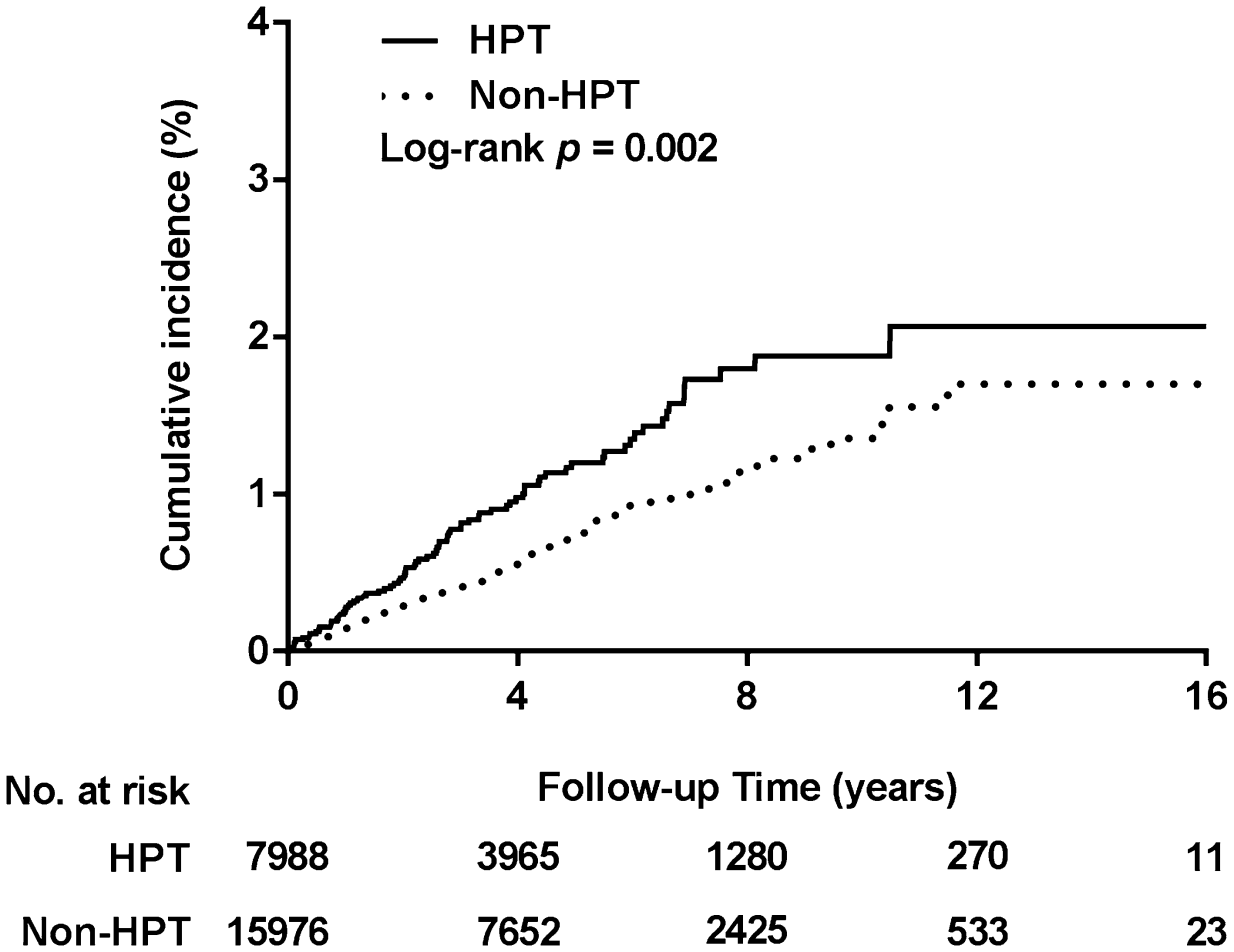
Cumulative incidence of voice dysfunction. ESRD, end-stage renal disease; HPT, hyperparathyroidism.

According to the Kaplan–Meier analysis results, the cumulative incidence of VD was significantly higher in the study cohort than in the comparison cohort (log-rank test, *p* = 0.002; Fig. 2). The results of Cox proportional hazard model analysis (Table 2) for VD risk demonstrated that HPT risk was approximately 1.6 times higher in the study cohort than in the comparison cohort (adjusted HR [95% CI] for the main model: 1.56 [1.17–2.09], adjusted HR [95% CI] for the full model: 1.59 [1.18–2.14]; both *p* = 0.003). In the sensitivity analyses, by alternately adding one comorbidity in the main model, we noted a considerable, constant risk of VD in the study cohort. Moreover, the subgroup analysis results revealed that interaction effects of HPT with sex, age, and comorbidities were nonsignificant for VD risk.

**Table 2:** Multivariable Cox Proportional Hazards Regression Model of the Association Between VD and Potential Risk Factors.

Variables	Adjusted HR (95% CI)	*p* value
**Multivariable regression analysis**		
Non-HPT	Reference	
HPT (main model^**†**^)	1.56 (1.17–2.09)	.003
HPT (full model^‡^)	1.59 (1.18–2.14)	.003
**Sensitivity analysis**^§^		
Main model + DM	1.56 (1.16–2.10)	.004
Main model + COPD	1.57 (1.17–2.10)	.003
Main model + LC	1.56 (1.17–2.09)	.003
Main model + CRS	1.56 (1.16–2.08)	.003
Main model + GERD	1.58 (1.18–2.11)	.002
**Subgroup analysis**		
Sex		
Female	1.35 (0.93–1.98)	.116
Male	1.94 (1.22–3.06)	.005
Age		
< 65	1.66 (1.20–2.29)	.002
≥ 65	1.22 (0.62–2.41)	.571
DM		
No	1.87 (1.25–2.78)	.002
Yes	1.19 (0.73–1.95)	.476
COPD		
No	1.68 (1.22–2.29)	.001
Yes	1.10 (0.50–2.41)	.813
LC		
No	1.51 (1.12–2.04)	.007
Yes	2.41 (0.73–7.92)	.147
CRS		
No	1.67 (1.24–2.24)	.001
Yes	0.37 (0.08–1.76)	.213
GERD		
No	1.75 (1.26–2.41)	.001
Yes	1.02 (0.52–2.00)	.953

HR, hazard ratio.

^**†**^Main model was adjusted for sex, age, urbanization level, and income level.

^‡^Full model was adjusted for sex, age, urbanization level, income level, and comorbidities.

^§^The models were adjusted for covariates in the main model as well as for each additional listed comorbidity.

### Part II—clinical study

We enrolled 60 hemodialysis patients with SHPT, comprising 25 men and 35 women (mean ± SD age 52.84 ± 11.52 [range 26–80] years; mean ± SD dialysis duration: 82.3 ± 24.2 [range 32–153] months; Table 3), half of whom complained of hoarseness or other laryngeal symptoms. Of them, 45 received total parathyroidectomy and autotransplantation.

**Table 3:** Characteristics of Enrolled Patients with ESRD (N = 60).

Characteristic	Number
**Sex**	
Male (n)	25
Female (n)	35
**Mean age (range)**	52.96 (26—80)
**Plasma level**	
Calcium (mg/dL)	10.00 ± 1.03
Phosphorous (mg/dL)	5.33 ± 1.34
iPTH (pg/mL)	1216.16 ± 767.03
**Aerodynamic**^†^	
MPT (s)	13.39 ± 5.68
s (s)	14.57 ± 7.30
z (s)	14.80 ± 7.30
**Acoustic**^‡^	
F0 (Hz)	177.99 ± 43.11
Jitter (%)	1.41 ± 0.84
Shimmer (dB)	0.38 ± 0.18
NHR	0.13 ± 0.03

iPTH, intact parathyroid hormone; F0, fundamental frequency; MPT, maximum phonation time; s, voiceless “s” produced without vibration of vocal folds; z, voiced “z” produced with vibration of vocal folds; NHR, noise-to-harmonics ratio.

^†^Aerodynamic parameters with stop watch.

^‡^Acoustic parameters in a Computerized Speech Laboratory.

In all included 60 patients, the mean ± SD plasma calcium, phosphorus, and iPTH levels were 10.00 ± 1.03 mg/dL, 5.33 ± 1.34 mg/dL, and 1216.16 ± 767.03 pg/mL, respectively. Regarding voice-associated parameters measured through CSL, the mean ± SD values of F0, jitter, shimmer, and NHR were 177.99 ± 43.11 Hz, 1.41% ± 0.84%, 0.38 ± 0.18 dB, and 0.13 ± 0.03, respectively; moreover, the mean ± SD MPT, “s” sound duration, and “z” sound duration were 13.39 ± 5.68, 14.57 ± 7.30, and 14.80 ± 7.30 s, respectively.

Of the 45 patients with SHPT who underwent total parathyroidectomy, 32 patients completed the preoperative and postoperative vocal evaluations through VSL and voice analysis.

There was no significant difference in laryngeal analysis between pre and post operation. No vocal fold lesion and no glottal gap was found among 32 patients before and after operation. The wave of vocal fold mucosa was normal and symmetric among these 32 patients. No supraglottic constriction was found in the study.

Table 4 lists the levels of calcium, phosphorus, and iPTH in these patients, all of which significantly decreased after surgery. In the objective vocal measurements, F0, jitter, and shimmer values demonstrated significant improvements postoperatively (F0: 178.0 ± 42.9 vs 236.5 ± 83.7 Hz, *p* = 0.01; jitter: 1.41% ± 0.84% vs 0.84% ± 0.35%, *p* = 0.047; shimmer: 0.38 ± 0.18 vs 0.24 ± 0.09 dB, *p* = 0.033). However, the preoperative and postoperative differences in perceptual evaluation and aerodynamic parameters, including MPT and duration of “s” and “z” sounds, were nonsignificant.

**Table 4:** Changes in Plasma Levels of SHPT Indicators, voice analysis, and Subjective Voice Assessment Results Before and 3 Months After Parathyroidectomy.

Variables	Preoperative	Postoperative	*p* value
**Plasma levels**			
Calcium (mg/dL)	10.00 ± 1.03	8.24 ± 1.44	.000*
Phosphorus (mg/dL)	5.33 ± 1.34	3.72 ± 1.55	.040*
iPTH (pg/mL)	1216.23 ± 767.05	88.73 ± 166.51	.000*
**Aerodynamic**^†^			
F0 (Hz)	178.03 ± 42.91	236.55 ± 83.79	.010*
Male	146.85 ± 34.67	167.09 ± 48.07	.267
Female	203.92 ± 38.24	231.44 ± 36.14	.178
MPT (s)	13.39 ± 5.64	14.48 ± 3.85	**.**922
s (s)	11.91 ± 5.70	13.17 ± 9.84	**.**988
z (s)	14.57 ± 7.25	14.93 ± 4.65	**.**744
**Acoustic**^‡^			
Jitter (%)	1.41 ± 0.84	0.84 ± 0.35	.047*
Shimmer (dB)	0.38 ± 0.18	0.24 ± 0.09	.033*
NHR	0.13 ± 0.03	0.13 ± 0.03	.342
**Perceptual**			
GRBAS Sum	0.464 ± 0.63	0.273 ± 0.47	.344

iPTH, intact parathyroid hormone; F0, fundamental frequency; MPT, maximum phonation time; s, voiceless “s” produced without vibration of vocal folds; z, voiced “z” produced with vibration of vocal folds; NHR, noise-to-harmonics ratio.

^†^Aerodynamic parameters through video strobe laryngoscopy.

^‡^Acoustic parameters through Computerized Speech Laboratory.

*Student *t* test.

## Discussion

ESRD-complicated with SHPT can influence the calcium and phosphorus balance, potentially leading to fine vocal muscle weakness and consequent functional vocal disturbances. Here, by using real-world and clinical data, we proved the aforementioned hypothesis and for the first time investigated the influence of HPT in voice change. Our results confirmed that SHPT in patients with ESRD is a predisposing factor for VD, such that ESRD patients with SHPT have an approximately 1.6 times higher VD risk than do ESRD patients without SHPT.

Insufficient water in the body may lead to decrease in the water content of the vocal cords and decrease in the secretion of the respiratory tract. The drying of the vocal cord surface may lead to changes in voice quality. Therefore, patients on hemodialysis often experience transient hoarseness at the end of dialysis^33–35^.

To clarify whether the VD was due to dialysis-associated dehydration or SHPT, we used a nationwide database and enrolled hemodialysis patients and divided them to cohorts of patients with and without HPT to investigate VD occurrence. Consequently, we could eliminate dialysis-associated VD as the causative mechanism, confirming HPT as a risk factor for VD.

Calcium blocks sodium ion flux through voltage-gated sodium channels. Thus, hypercalcemia results in inhibition of depolarization and impairment of action potential generation in neurons and muscle cells^36^. Moreover, hypercalcemia can damage the recurrent laryngeal nerve and intrinsic laryngeal muscle, and this can result in dysphonia. These findings support our hypothesis that iPTH influences vocal production.

On the basis of Taiwan Renal Registry Data System, a large-scale study reported that a serum phosphorus level of ≥ 6.5 mg/dL was associated with increased mortality^37^. However, a significant imbalance of such electrolytes can be life-threatening.

Thus, when hemodialysis patients report changes in their voices, clinicians must include SHPT in the differential diagnosis. If in doubt, plasma phosphorus, calcium and iPTH levels could be estimated. Taken together, these steps could facilitate early detection of SHPT.

In our current clinical study, SHPT was found to significantly increase plasma calcium, phosphate, and iPTH levels before surgery. In the voice analysis after surgery, only the acoustic indices F0, jitter, and shimmer demonstrated significant differences. Studies have reported that abnormal vocal cord lesions typically increase jitter and shimmer. If Jitter exceeds the threshold of 1% and shimmer exceeds the threshold of 0.35 dB, abnormal performance is regarded to have occurred^38^. In the current study, the mean preoperative jitter was 1.41% (i.e., > 1%), but normalized to 0.84 postoperatively; similarly, the mean preoperative shimmer was 0.38 dB (i.e., > 0.35), but normalized to 0.24 dB postoperatively. Therefore, we speculated that SHPT mainly causes changes in the acoustic indices jitter and shimmer in the voice analysis, with the thresholds identical to those reported previously; this is a clinically significant observation. Jitter and shimmer represent variations in vocal fold vibrations. The change in vibration may result from changes in vocal muscle strength. An improvement (i.e., decrease) in the acoustic parameters produces a more harmonic voice.

This study had several strengths. First, we analyzed a large amount of data from numerous dialysis patients. Second, we verified the real-world evidence regarding HPT as an independent risk factor for VD. We also analyzed the clinical data of ESRD patients with and without SHPT and even evaluated their data before and after parathroidectomy to further verify the relationship between SHPT and voice disorders. Consequently, we provided reference indexes for clinicians when performing vocal examination through VSL and voice analysis.

Our study however has limitations related to real-world data investigations. First, the NHIRD does not provide patient data for clinical symptoms, laboratory data, and VSL results; therefore, we could not determine the VD severity in the included patients. Nevertheless, in our clinical study, we performed clinical examinations to obtain the data unavailable in the NHIRD. Although only ESRD patients with SHPT were enrolled in this study, our research could still provide clinical evidence supporting our hypothesis. In future studies, laryngeal electromyography (LEMG) should be applied to evaluate laryngeal muscle activity.

Based on our current findings, the use of VSL examinations and voice analysis is ideal for analyzing ESRD patients with voice disturbances. Moreover, the abnormal acoustic indices could be used for speculating whether SHPT may be an underlying cause of VD. Nevertheless, future studies should confirm the accuracy and sensitivity of these factors. Moreover, voice changes after nonsurgical treatment of SHPT warrant further research.

In conclusion, by using real-world and clinical patient data, we confirmed that SHPT is a predisposing factor for VD. In addition, the comparative results before and after surgery indicated that the acoustic indices jitter and shimmer in voice analysis might indicate the influence of SHPT on the voice of patients with ESRD. Thus, when a clinician encounters a patient with ESRD complaining of voice disturbances, they must consider including SHPT in the differential diagnosis to facilitate early detection and treatment of the underlying problem.

## Supplementary Information

Supplementary Infomation 1.

Supplementary Infomation 2.

Supplementary Infomation 3.
